# The influence of expertise on brain activation of the action observation network during anticipation of tennis and volleyball serves

**DOI:** 10.3389/fnhum.2014.00568

**Published:** 2014-08-01

**Authors:** Nils Balser, Britta Lorey, Sebastian Pilgramm, Tim Naumann, Stefan Kindermann, Rudolf Stark, Karen Zentgraf, A. Mark Williams, Jörn Munzert

**Affiliations:** ^1^Institute for Sport Science, University of GiessenGiessen, Germany; ^2^Bender Institute of Neuroimaging, University of GiessenGiessen, Germany; ^3^Institute of Sport and Exercise Sciences, Westfälische Wilhelms-University of MünsterMünster, Germany; ^4^Centre for Sports Medicine and Human Performance, Brunel University LondonLondon, UK

**Keywords:** sports-related anticipation, motor expertise, cerebellum, superior parietal lobe, functional magnetic resonance imaging

## Abstract

In many daily activities, and especially in sport, it is necessary to predict the effects of others' actions in order to initiate appropriate responses. Recently, researchers have suggested that the action–observation network (AON) including the cerebellum plays an essential role during such anticipation, particularly in sport expert performers. In the present study, we examined the influence of task-specific expertise on the AON by investigating differences between two expert groups trained in different sports while anticipating action effects. Altogether, 15 tennis and 16 volleyball experts anticipated the direction of observed tennis and volleyball serves while undergoing functional magnetic resonance imaging (fMRI). The expert group in each sport acted as novice controls in the other sport with which they had only little experience. When contrasting anticipation in both expertise conditions with the corresponding untrained sport, a stronger activation of AON areas (SPL, SMA), and particularly of cerebellar structures, was observed. Furthermore, the neural activation within the cerebellum and the SPL was linearly correlated with participant's anticipation performance, irrespective of the specific expertise. For the SPL, this relationship also holds when an expert performs a domain-specific anticipation task. Notably, the stronger activation of the cerebellum as well as of the SMA and the SPL in the expertise conditions suggests that experts rely on their more fine-tuned perceptual-motor representations that have improved during years of training when anticipating the effects of others' actions in their preferred sport. The association of activation within the SPL and the cerebellum with the task achievement suggests that these areas are the predominant brain sites involved in fast motor predictions. The SPL reflects the processing of domain-specific contextual information and the cerebellum the usage of a predictive internal model to solve the anticipation task.

## Introduction

One can think of many different situations where it is a crucial skill to anticipate what is going to happen next. For example, a car driver has to anticipate whether a person approaching a pedestrian crossing is going to cross the street or not, surgeons have to be aware of the upcoming actions of their colleagues in the operating theater, whereas a goalkeeper in soccer has to identify the shoot direction of a penalty taker as soon as possible. For the last example, researchers have shown that the ability to anticipate the effect of the observed actions is paramount to successful performance (Savelsbergh et al., 2002; Williams et al., 2011). Fast ball sports, like tennis or volleyball, provide perfect tasks to investigate the processes underlying the anticipation of action effects as well as the influence of the athlete's prior perceptual and motor experience. In these kinds of sports, one can find many situations where athletes are under enormous time pressure and have to decide on an appropriate response even before the opponent has finished his/her action, as can be seen during the tennis return of serves with above 200 km/h for example (Williams et al., 2011). Over the last few decades, numerous researchers have shown that experts outperform novices when anticipating their opponents' actions (e.g., Singer et al., 1996; Abernethy et al., 2001; Rowe and McKenna, 2001; Williams et al., 2002; Cañal-Bruland et al., 2011; for a review, see Williams et al., 2011). The results indicate that experts rely on information visually conveyed by the kinematics of their opponent's action ahead of a key event such as ball-racket or ball-foot contact, (Abernethy and Russell, 1987; Aglioti et al., 2008; Huys et al., 2008; Williams et al., 2009; Urgesi et al., 2011).

On the neural level, the action observation network (AON) is supposed to play a crucial role in the perception of another person's action. This network comprises all brain areas that are activated by the mere observation of actions (Cross et al., 2009). A meta-analysis of 104 studies revealed enhanced activation during the observation of hand movements in the inferior frontal gyrus (BA 44/45), the dorsal premotor cortex (dPMC), the inferior parietal cortex (IPL), the superior parietal cortex (SPL), the inferior parietal sulcus (IPS), the primary somatosensory cortex (SI), the posterior medial temporal gyrus (pMTG), the fusiform face/body area (FFA/FBA), and the visual area V5 (Caspers et al., 2010). Furthermore, an activation of the cerebellum during action observation has been reported by numerous researchers (Buccino et al., 2004; Gallagher and Frith, 2004; Gazzola et al., 2007; Gazzola and Keysers, 2009; Pilgramm et al., 2010; Molenberghs et al., 2012; Balser et al., 2014). These data indicate that the cerebellum is part of the AON as well (Calvo-Merino et al., 2006). The AON, however, seems to be a dynamic and experience-related system. In this regard, Calvo-Merino et al. (2006) examined male and female ballet dancers who were observing gender-specific dance videos. They found stronger activation in the cerebellum and other areas within the AON, namely the dPMC and the IPS, when dancers saw dancing steps from their own motor repertoire compared to moves of the other gender with whom they had only visual familiarity. These results indicate that motor expertise has an influence on the neural processes in the cerebellum and the whole AON.

One of the various functions that are discussed for the AON is the anticipation of the consequence of an action (Gazzola and Keysers, 2009; Zentgraf et al., 2011) which might be the next action step or the environmental effect of an action. Thus, activation within this network is associated with anticipation in everyday actions (Stadler et al., 2012; Avenanti et al., 2013) and in sports-related actions (Wright et al., 2010, 2011; Abreu et al., 2012; Bishop et al., 2013; Balser et al., 2014). The specific role of the SPL and the cerebellum during an anticipation task was reported in a study conducted in our laboratory (Balser et al., 2014). During anticipation, tennis experts showed an enhanced activation in IFG and SPL, as well as a strong activation increase in numerous parts of the cerebellum, more precisely in Crus I, Crus II, Lobule VII and Lobule VIII. Furthermore, the data revealed that the neural activation of the SPL and parts of the cerebellum co-varies linearly with anticipation performance. The latter results indicate that posterior parietal and cerebellar areas of the AON are actually involved in the anticipation of action effects, as the performance-related activation increase was specific to these areas (Balser et al., 2014). A potential role of the SPL during action prediction is the storage of internal models and perceptual-motor representations (Winstein et al., 1997; Wolpert et al., 1998a; Miall, 2003; Rizzolatti and Matelli, 2003). As posterior parietal areas, the cerebellum is described as a principal brain structure for the storage of internal forward models that predict the outcome of actions (Wolpert et al., 1998b; Imamizu et al., 2000; Bastian, 2006; Miall and King, 2008; Synofzik et al., 2008).

One of the shortcomings that apply for most of the previous studies examining expert-novice differences is related to the population investigated. In many studies experts are compared to novices that did not only fail to exhibit expertise in the particular sports that is investigated, but that also differ in principle with respect to any anticipation experience (Wright et al., 2010, 2011; Abreu et al., 2012; Balser et al., 2014). In this case, differences between experts and novices could be caused many different factors such as unfamiliarity with the task which requires attentional resource allocation, decision-making under time pressure etc. In the present study, these problems have been addressed by comparing expert athletes from two different sports that both imply anticipation expertise, but expertise only for a specific sport. Therefore, this study differs markedly from prior studies as we examine two different expert groups during the anticipation of an opponent's action in tennis and volleyball to better understand the role of the AON and of the cerebellum. This approach allows us to examine in a within-subject design whether anticipation of action effects recruits areas of this network depending on the very specific representations of the observed movement in the subject's personal motor repertoire.

We applied a 2 × 2 design with two different expertise groups (between-subject condition: tennis experts vs. volleyball experts) anticipating serves from two types of sports (within-subject condition: tennis serves vs. volleyball serves). All participants watched video clips of serves in their particular sport of expertise as well as in the sport with which they had only little experience. Thus, we compared two expert groups who both had exceptional anticipation skills in their specific domain of expertise but who were at the same time novices in the other sport. In both groups, the instruction was to anticipate the direction of the serves (left vs. right) that were occluded at the moment of ball–racket or ball-hand contact respectively. Based on prior studies on perceptual-motor representations, we expected stronger activation in areas of the AON and the cerebellum in athletes with high expertise compared with novices. Second, we expected a performance-dependent activation increase in motor experts which co-varies with the task performance within the AON that were suggested to contain well-defined perceptual-motor representations. Likely candidates are posterior parietal and cerebellar structures, as these areas are associated with the storage of internal models that support predictive motor control.

## Materials and methods

### Participants

All thirty one participants were right-handed according to the Edinburgh Handedness Inventory (Oldfield, 1971). They had normal or corrected to normal vision and had not reported any history of psychiatric or neurological disorders or current use of psychoactive medication. The sample consisted of 15 tennis experts (8 female, mean age = 23.87, *SD* = 5.26) and 16 volleyball experts (8 female, mean age = 25.69, *SD* = 4.19). All thirty one experts were playing in one of the four highest level leagues in Germany in their respective sport and had experience only at a recreational level in the sport in which they were not an expert. Tennis experts had played an average of 461 (*SD* = 222) tournament matches in a mean time period of 16.67 (*SD* = 5.94) years, volleyball experts had a mean experience of 12.69 (*SD* = 5.33) years and 343 (*SD* = 215) matches. Both groups did not differ significantly in any of the reported characteristics. Participants were paid and gave their informed written consent in accordance with the Declaration of Helsinki. The study was approved by the local ethics committee (LEK FB06, 2011–0026) at the lead institution.

### Stimuli

**Figure 1:**
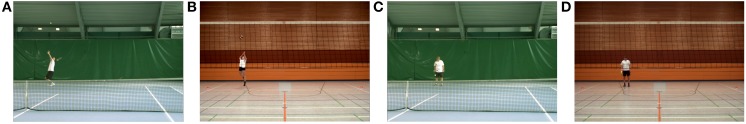
**Screenshots of all four experimental conditions**. Each of the 128 video clips lasted 2.9–4.6 s. **(A)** Male tennis player performing a tennis serve (*Tennis Anticipation* condition). **(B)** Female volleyball player performing a volleyball serve (*Volleyball Anticipation* condition). All serve sequences were stopped at ball–racket respective ball–hand contact. **(C)** Female tennis player bouncing the ball with her racket (*Tennis Observation* condition). **(D)** Male volleyball player bouncing the ball with his hand (*Volleyball Observation* condition).

Participants observed 128 stimulus videos with a duration ranging from 2.9 to 4.6 s. Half of them showed tennis and volleyball serves performed by a male and a female right handed model from each sport that were playing on the same level as the corresponding expertise group in our study. The tennis as well as the volleyball serves were all stopped at ball-racket or ball-hand contact respectively. For the videos of the tennis serves, the camera was placed right before the baseline at a position that is typical for a player waiting to return the opponent's serve (cf. Figure 1A). To simulate the situation of a volleyball player waiting to receive an opponent's serve, for the volleyball serves the camera was positioned 6 m behind the net in the middle of the field (cf. Figure 1B). One half of the 32 video clips from each sport showed serves to the left-hand corner and one half showed serves to the right-hand corner of the volleyball field or to the right service box of the tennis court respectively. The remaining 64 video clips displayed the two models of both sports bouncing a tennis ball with their racket respectively a volleyball with their right hand standing at the baseline (cf. Figures 1C,D). All stimuli were recorded using a Basler avA 1600—50 gc (Basler AG, Ahrensburg, Germany) video camera with a sampling rate of 35 fps.

The 128 video clips were presented at a resolution of 1024 × 768 pixels with a PC running Presentation software (Version 12.9, Neurobehavioral Systems, Albany, USA) and projected onto a screen behind the scanner so that the participants could watch them via a mirror attached to the head coil (visual field 188 mm in the horizontal and 168 mm in the vertical plane, rectangular aperture; visual angle approximately 18° horizontal and 11° vertical).

### Task

Participants had to respond to four different conditions. In the *Tennis Anticipation* condition, they watched tennis serves and were asked to anticipate the direction of the observed serve and subsequently indicate the perceived flight direction of the ball. In the *Volleyball Anticipation* condition, participants watched volleyball serves with the same instruction. In both anticipation conditions, the response was given by pressing the left or right button on a two-button response box. The left button indicated a ball flying to the left-hand corner and the right button a ball flying to the right-hand corner. To control for effects due to visual stimulation and the observation of biological movements, we added a *Tennis Observation* and a *Volleyball Observation* conditions including the same two models in the same visual setting without any instruction for explicit anticipation. The task in these two observation only conditions was to observe the models bouncing the ball with their racket or their hand respectively and to press the left or right button immediately after the video. The instruction text indicated which button to press before each video. All responses in this study included motor reactions after the respective observation condition. The ratio of correct left and right reactions was balanced across all four conditions.

### Procedure

Participants were given instructions for the experimental conditions illustrated with sample videos and figures. Before the start of the fMRI experiment, participants completed a short training session with two videos for each experimental condition to ensure their full understanding of the tasks. These videos were not used in the fMRI session. While lying in the scanner, participants had to complete 128 trials resulting in a total duration of 34 min for the whole experiment. The order of the trials was randomized for each participant. Every trial started with a black screen for 1 s, an instruction for 3 s and a fixation cross for another 5 s. The following presentation of the video sequence lasted 2.9–4.6 s. The screen turned blank instantaneously after the video presentation. The participants were instructed to give their response as quickly as possible by pressing the left or the right button on the response box with the index and middle finger of their right hand. When a button was pressed, the given response was displayed on the screen for the rest of the available response time (3 s). During the whole experiment, participants did not receive any feedback on their performance.

### Behavioral data acquisition and analysis

In each of the four experimental conditions, both correct answers and response times (defined as the time between the end of the video stimulation and the button press) were analyzed with SPSS (Version 19, IBM, Chicago, USA). To investigate the influence of expertise on the number of correct responses, a 2 × 2 mixed ANOVA with Anticipation task (*Tennis Anticipation* vs. *Volleyball Anticipation*) as repeated measures within-subject factor and Domain of expertise (tennis experts vs. volleyball experts) as between-subject factor was performed. The same computation was employed for the response times. Additionally, *t*-tests within each group assessed whether the number of correct responses in the *Tennis* and the *Volleyball Anticipation* condition were significantly above chance level.

### fMRI data acquisition and preprocessing

The fMRI data were acquired using a 1.5 Tesla whole body scanner (Siemens symphony, Erlangen, Germany) with a standard head coil. The structural images consisted of 160 T1-weighted sagittal images (slice thickness = 1 mm, *TR* = 1.99 s, *TE* = 4.18 ms, field of view = 250 × 250 mm, base resolution = 256 × 256, orientation = sagittal). During the experiment, a total of 816 T2^*^-weighted images were collected using a gradient echo-planar-imaging sequence (number of slices = 25, slice thickness = 5 mm, gap = 1 mm, *TA* = 100 ms per slice, *TR* = 2.5 s, *TE* = 55 ms, flip angle = 90°, field of view = 192 × 192 mm, matrix size = 64 × 64). The axial slices recorded during the EPI sequence were oriented parallel to the AC–PC line. The onsets of the video clips were jittered within an interval between ± ½ TR to realize a better sampling of the HRF function.

Functional data were processed and analyzed using SPM8 (Wellcome Department of Cognitive Neurology, London, UK). The 816 volumes were realigned and unwarped, slice-time corrected, and normalized into Montreal Neurological Institute (MNI) space. Finally, data were smoothed with 9-mm Gaussian isotropic filter as recommended by Worsley (2007). Furthermore, a movement correction was employed to reduce the impact of rapid head movements by the usage of in-house software. The detection of outlier volumes was based on a comparison of each volume with its two neighbors in a motion-corrected time series. This procedure was done by calculating the mean squared differences to the previous and the next volume. The smaller difference was used as the outlier score for each volume. Scores were thresholded using Hubert and van der Veeken's (2008) method of calculating a skewness-corrected interquartile range. To threshold outlier scores, the range was multiplied by 1.5 and added to the 75th percentile. Later on, the correction of outlier volumes was done during the first-level analysis by the usage of an additional regressor for each odd volume.

For the cerebellar data, a specific normalization method was applied to allow a more accurate localization of activation within the small structures of the cerebellum. Because of the low contrast within the cerebellum in the 152 ICBM template (MNI space), a standard whole-brain normalization as used in SPM8 leads to a large spatial variance between participants (Diedrichsen, 2006). Therefore, we used the template of the SUIT toolbox for SPM8 (Version 2.5.3, Institute of Cognitive Neuroscience, London, UK), which is based on the average cerebellar anatomy of 20 participants. This procedure preserved the fine details of the cerebellum and improved the intersubject alignment compared to the standard normalization (Diedrichsen, 2006). In a first step, the automatic isolation algorithm provided by the toolbox was used to segregate the cerebellum and the brainstem. If necessary, the isolation maps were corrected manually based on anatomical information and were then normalized to the SUIT template via a nonlinear transformation. The resultant deformation maps were used to normalize the functional images of each participant. Contrary to the whole brain data, in which normalization and the ensuing smoothing were performed before the first-level analysis, in the SUIT normalization, these steps were conducted after the functional data had been analyzed on the single-subject level. On the second-level, the whole-brain and the cerebellar data were analyzed in exactly the same way.

### Data analysis

The first-level analysis was computed for each participant separately on the basis of the general linear model (GLM). The signal was convoluted using the hemodynamic response function (HRF). The video observation of each trial in the four conditions was covered by this HRF matching the length of the video. Functional data were high-pass filtered with a cutoff of 128 s to remove slow signal changes. The correct and incorrect trials of the four different experimental conditions (*Tennis Anticipation*, *Volleyball Anticipation*, *Tennis Observation*, and *Volleyball Observation*) as well as the instructions and the responses were entered into the model. Furthermore, six parameters resulting from the movement correction were added to the GLM as covariates. Autoregressive processing was applied to account for serial correlations.

In the second-level analysis, one-sample and two-sample *t*-tests were conducted. To identify brain activation correlated with the anticipation performance irrespective of the expertise of the participants, we introduced the parameter “percentages of correct responses in both anticipation conditions” as a parameter to the contrast *Tennis and Volleyball Anticipation* > *Tennis and Volleyball Observation* for all 31 participants. To investigate the role of expertise during effect anticipation, the contrast (*Expertise Anticipation* > *Expertise Observation*) > (*Novice Anticipation* > *Novice Observation*) was analyzed with a two-sample *t*-test in both groups. In this contrast the common activation of both groups during the anticipation of serves of the own expertise sport compared to the sport the participants had no experience with was identified, whereas differences due to different stimuli were controlled by considering the control conditions (*Expertise Observation* and *Novice Observation*). **For a comparison of the tennis experts anticipating tennis serves with the volleyball experts anticipating volleyball serves, please see the Supplementary Material**. Additionally, we fed the covariate “percentages of correct responses in the expertise anticipation condition” into the contrast (*Expertise Anticipation* > *Expertise Observation*) > (*Novice Anticipation* > *Novice Observation*) to eliminate the influence of the anticipation performance in the respective expertise sport on the activation in areas of the AON identified by this contrast. More precisely, this additional regressor in the design matrix specified the subject-specific information of correct responses made during the different tasks. The respective contrast then focuses on neural activation due to expertise during an anticipation task, partialing out activation due to the correct responses made. Furthermore, in a second parametric analysis, we introduced the percentages of correct responses in the expert anticipation condition as a further covariate to the contrast *Expertise Anticipation* > *Expertise Observation* for all 31 participants to investigate whether AON activation in the expertise sport is correlated with the anticipation performance. This analysis focuses on the specific effects of the covariate as the respective parameter estimate represents the magnitude of the correlation between anticipation-specific activation and the number of correct responses made.

With respect to our research questions, we were particularly interested in brain activation within the areas of the AON, and we expected to find activation differences within these areas depending on expertise. Therefore, we examined a small-volume correction with a priori defined search volumes in the AON for all contrasts comparing the respective expertise and novice anticipation conditions of the athletes. The selection of these regions of interest (ROIs) was based on the results of Caspers et al.'s (2010) meta-analysis and included the inferior parietal lobe (IPL), the superior parietal lobe (SPL), the dorsal and ventral premotor cortex (dPMC and vPMC), the supplementary motor area (SMA), the somatosensory cortex (S1), and the inferior frontal gyrus (IFG). Because Caspers et al.'s (2010) meta-analysis did not include the cerebellum, we chose ROIs in the cerebellum that had been reported to be activated during the execution (e.g., Dimitrova et al., 2006; Schmahmann et al., 2009), the observation (e.g., Sokolov et al., 2010) and the anticipation (Balser et al., 2014) of actions. These regions were Lobules I-IV, V, VI, VII, and VIII, as well as Crus I and Crus II. The cerebellar masks were based on the probabilistic atlas of the cerebellum provided by Diedrichsen et al. (2009), whereas the masks of the cerebral cortex were based on cytoarchitectonic data (Eickhoff et al., 2005). All masks for this ROI analysis were created using FSL software (Smith et al., 2004) and included voxels with an at least 50% probability of being part of the specific regions. The statistical threshold for the ROI analysis was set at *p* = 0.05 (FWE-corrected). To examine whether the expertise and the novice anticipation condition are associated with differential attention-related processes, for the contrast (*Expertise Anticipation* > *Expertise Observation*) > (*Novice Anticipation* > *Novice Observation*), we compared activation in the frontal eye field (FEF) in both anticipation conditions in a *post-hoc* analysis. Therefore, we used 10-mm spheres around the MNI coordinates suggested by Heinen et al. (2013) (MNI coordinates right FEF: 31, 1, 58; MNI coordinates left FEF: −31, −3, 57) with the same statistical threshold (*p* = 0.05, FWE-corrected).

## Results

### Behavioral data

**Figure 2:**
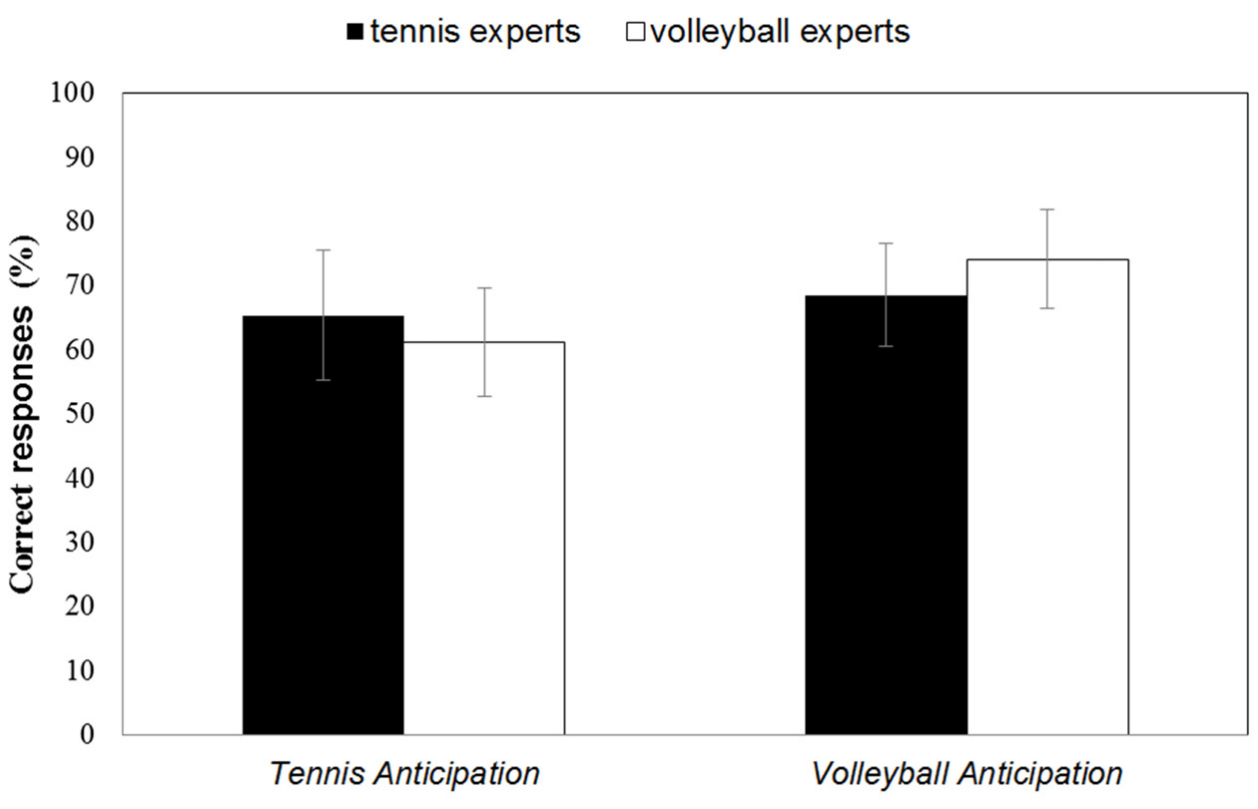
**Mean percentage of correct responses in the Tennis Anticipation and the Volleyball Anticipation condition of the tennis experts and the volleyball experts**. Bars represent *SD*.

In the tennis anticipation condition, tennis experts gave correct answers on an average of 65.42% (*SD* = 10.12) of trials, while volleyball experts reported correct responses on 61.14% (*SD* = 8.46) of trials. When anticipating volleyball serves, volleyball experts had a mean accuracy score of 74.19% (*SD* = 7.76), whereas tennis experts responded correctly on an average of 68.54% (*SD* = 8.05). In both groups the number of correct responses was significantly above chance level for the anticipation of the tennis [*t*_tennis experts(14)_ = 5.90, *p* < 0.001; *t*_volleyball experts(15)_ = 5.26, *p* < 0.001] as well as for the volleyball serves [*t*_tennis experts(14)_ = 8.92, *p* < 0.001; *t*_volleyball experts(15)_ = 12.59, *p* < 0.001]. A 2 (Domain of expertise) × 2 (Anticipation task) ANOVA with repeated measures for the last factor revealed a significant interaction between both factors, *F*_(1, 29)_ = 5.66, *p* = 0.024, η^2^ = 0.163 (with higher scores for correct anticipation in each sport for the respective expert group compared to the less experienced group), as well as a significant main effect on the Anticipation task, *F*_(1, 29)_ = 14.76, *p* = 0.001, η^2^ =0.337 (higher scores for correct anticipation in volleyball) (cf. Figure 2). No significant main effect was reported for the between-subject factor Domain of expertise, *F*_(1, 29)_ < 1, ns.

Tennis experts had a mean response time of 513 ms (*SD* = 211) in the tennis anticipation condition and 574 ms (*SD* = 174) in the volleyball anticipation condition. For the volleyball experts the average response times were 641 ms (*SD* = 146) in the tennis anticipation condition and 608 ms (*SD* = 148) in the volleyball anticipation condition. A 2 (Domain of expertise) × 2 (Anticipation task) ANOVA with repeated measures for the last factor revealed a significant interaction between both factors, *F*_(1, 29)_ = 9.56, *p* = 0.004, η^2^ = 0.248 (faster response of both expertise groups in their respective expertise sport). Neither a significant main effect for the between-subject factor Domain of expertise, *F*_(1, 29)_ = 1.80 *p* = 0.190, η^2^ = 0.058, nor for the within-subject factor Anticipation task, *F*_(1, 29)_ < 1, ns, was reported.

In the ball-bouncing conditions (*Tennis Observation* and *Volleyball Observation*), participants were asked to press either the left or right button depending on the instruction received before each video. In both groups 99% of the responses were correct, indicating that all participants had maintained attention in the *Tennis Observation* and the *Volleyball Observation* condition during the whole experiment.

### fMRI data

The study was designed to identify the influence of motor expertise on the brain activation during the anticipation of action effects. Based on the results of our previous study (Balser et al., 2014), we expected stronger activation in areas of the AON when participants anticipated the effects of actions within their domain of expertise. Therefore, in all 31 participants the brain activation during the anticipation in the respective expertise condition was contrasted with the condition the participants had no experience with. To eliminate the influence of the anticipation performance in the respective expertise sport on the activation in areas of the AON identified by this contrast, a covariate “percentages of correct responses in the expertise anticipation condition” was introduced. In a further step, we performed two parametric analyses to investigate whether activation in areas of the AON was correlated with anticipation performance irrespective of expertise or with the anticipation performance in the expertise sports.

#### Expertise-related differences in the activation of the AON during anticipation

**Figure 3:**
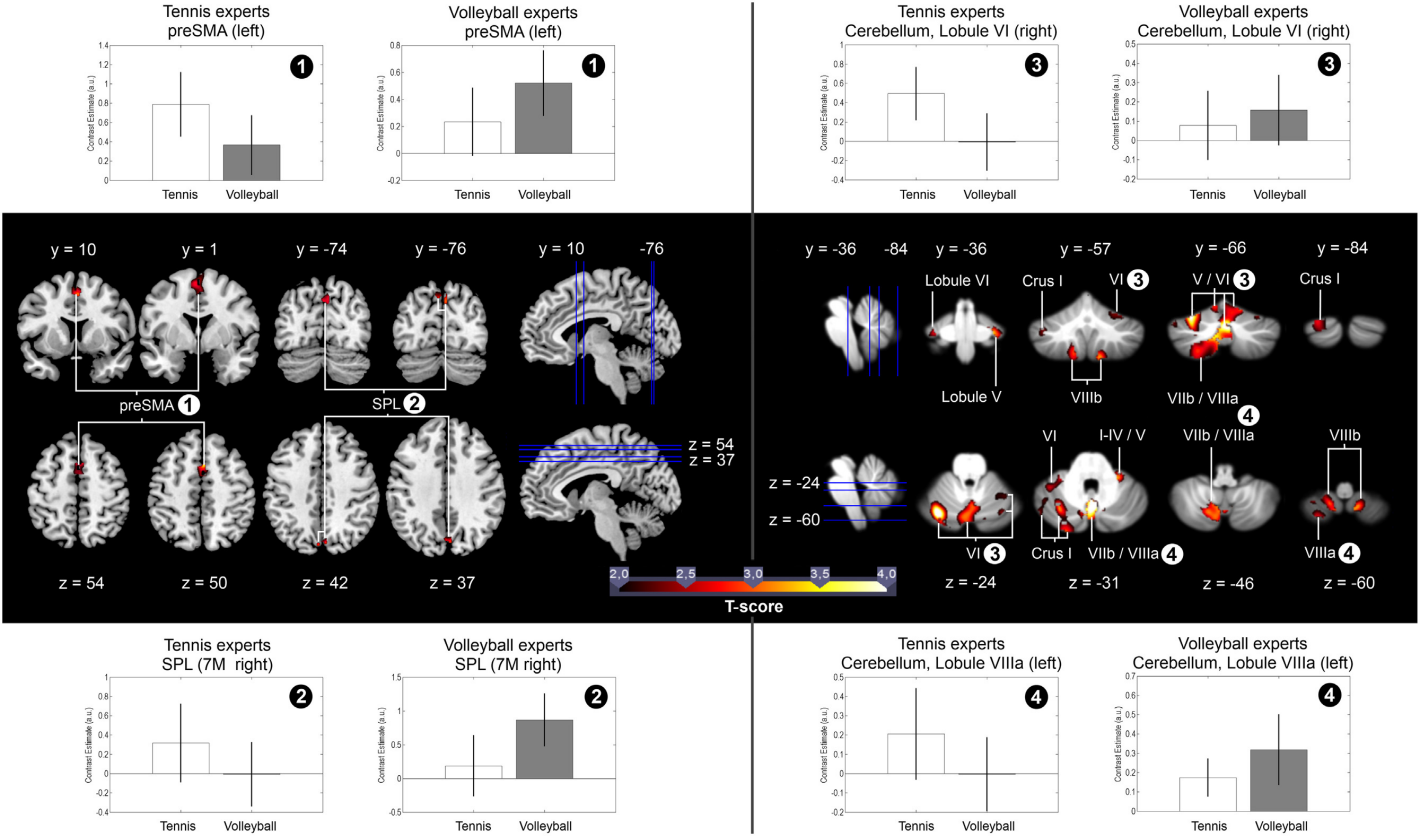
**In the middle of the figure: Significant brain activation in all 31 participants for the contrast (*Expertise Anticipation* > *Expertise Observation*) > (*Novice Anticipation* > *Novice Observation*)**. The blue vertical and horizontal lines indicate the slice positions. T maps were thresholded at *t* = 2.00 (*p* < 0.05, FWE-corrected). Activation is rendered on a high-resolution T1 template (“colin brain”) as well as on the cerebellar SUIT template (Diedrichsen, 2006). Upper and lower part of the figure: Mean percent signal changes and standard errors in the preSMA, the SPL, and in Lobule VI and VIIIa of the cerebellum for the contrasts *Tennis Anticipation* > *Tennis Observation* and *Volleyball Anticipation* > *Volleyball Observation*, separated for both expertise groups. The signal changes were calculated by means of the SPM toolbox rfxplot (Gläscher, 2009; http://rfxplot.sourceforge.net).

Based on the results of a previous study (Balser et al., 2014), we examined the hypothesis that anticipating the effect of actions, the observer has expertise for, is correlated with stronger activation of AON areas. To identify these differences, we compared brain activation during the anticipation of serves in the respective expertise sport with anticipation in the type of sport the participants were novices for. Each anticipation condition was contrasted first with the ball bouncing condition of the same sport resulting in the contrast (*Expertise Anticipation* > *Expertise Observation*) > (*Novice Anticipation* > *Novice Observation*) for all 31 participants. Because the ball-bouncing control conditions contained the observation of biological movements of the same players in the identical visual settings, the results of this contrast reflect brain activation due to expertise-related anticipation and not to the mere observation of biological motion or the button press. The within-subject ROI analysis revealed higher activation for anticipation in the experts for the superior parietal lobe (SPL), the presupplementary motor area (preSMA), as well as for broad sections of the cerebellum: Crus I, Crus II, Lobule I-IV, Lobule V, Lobule VI, Lobule VIIb, Lobule VIIIa and VIIIb, Lobule IX, and Lobule X (*p* < 0.05, FWE-corrected) (cf. Figure 3). The opposite contrast (*Novice Anticipation* > *Novice Observation*) > (*Expertise Anticipation* > *Expertise Observation*) did not reveal any significant brain activation for the novice anticipation condition compared to the expertise anticipation condition. When the influence of different anticipation performance scores in both sports was eliminated by introducing the covariate “percentage of correct responses in the expert anticipation condition” (*M* = 70.06%, *SD* = 9.94), the contrast (*Expertise Anticipation* > *Expertise Observation*) > (*Novice Anticipation* > *Novice Observation*) resulted in activation in the same activation sites, as well as in an additional activation within the IFG. All results are summarized in Table 1.

**Table 1:** **Brain areas identified by the comparison of the respective expertise anticipation condition with the corresponding novice anticipation condition in all 31 participants**.

	**L/R**	***X***	***Y***	***Z***	***t*-value**	**SUIT**	Co-variate^*^
**(EXPERTISE ANTIC. > EXPERTISE OBS.) > (NOVICE ANTIC. > NOVICE OBS.)**							
preSMA	R	3	11	50	3.71		✓
preSMA	L	−3	−1	62	3.33		✓
SPL (7 PC)	L	−3	−79	41	3.49		✓
SPL (7 M)	R	6	−76	38	3.19		✓
SPL (7 M)	L/R	0	−73	32	3.21		✓
Cerebellum, Crus I	L	−30	−72	−25	4.37	✓	✓
Cerebellum, Crus I	L	−4	−78	−27	3.11	✓	✓
Cerebellum, Crus II	L/R	0	−72	−31	3.95	✓	✓
Cerebellum, Lobule I-IV	R	26	−34	−35	3.27	✓	✓
Cerebellum, Lobule V	R	28	−38	−33	3.42	✓	✓
Cerebellum, Lobule VI	L	−30	−70	−21	5.13	✓	✓
Cerebellum, Lobule VI	R	2	−62	−29	4.41	✓	✓
Cerebellum, Lobule VI	R	8	−70	−13	3.57	✓	✓
Cerebellum, Lobule VIIb	L	−14	−68	−43	3.57	✓	✓
Cerebellum, Lobule VIIb	R	2	−66	−31	4.32	✓	✓
Cerebellum, Lobule VIIIa	L	−8	−66	−39	3.52	✓	✓
Cerebellum, Lobule VIIIa	R	4	−62	−31	4.58	✓	✓
Cerebellum, Lobule VIIIb	L	−8	−64	−41	3.55	✓	✓
Cerebellum, Lobule VIIIb	R	14	−58	−61	3.31	✓	✓

*Each anticipation condition was contrasted with the ball bouncing condition of the same sport (Expertise Anticipation > Expertise Observation) > (Novice Anticipation > Novice Observation)*.

* *Same activation found when a covariate “percentages of correct responses in the expert anticipation condition” was introduced. MNI coordinates, p < 0.05, FWE-corrected, ROI analysis, ROI masks thresholded at 50%, for all ROI masks used for this analysis see Section Data Analysis at page 4*.

#### Performance-related differences in the activation of the AON during anticipation

**Figure 4:**
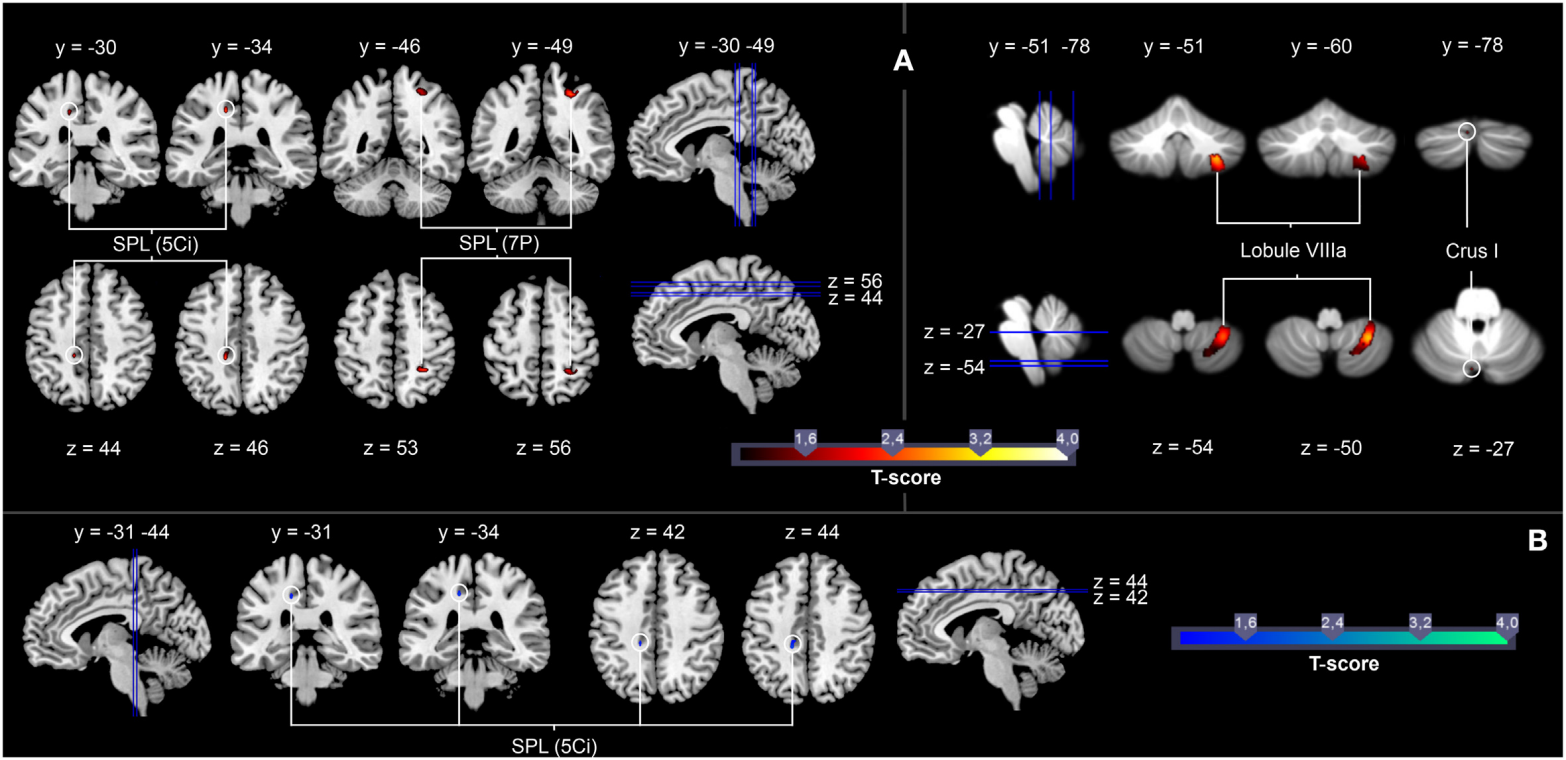
**(A)** Brain areas showing significantly stronger activation as a function of the number of correct responses for the contrast *Tennis* and *Volleyball Anticipation* > *Tennis* and *Volleyball Observation* in all 31 participants (red marks). **(B)** Brain areas showing significantly stronger activation as a function of the number of correct responses in serve anticipation in the expertise sport for the contrast *Expertise Anticipation* > *Expertise Observation* in all 31 participants (blue marks). T maps were thresholded at *t* = 1.00 (*p* < 0.05, FWE-corrected). Activation is rendered on a high-resolution T1 template (“colin brain”) as well as on the cerebellar SUIT template (Diedrichsen, 2006).

As we expected a performance-dependent activation increase irrespective of expertise sport within areas that are suggested to contain motor skill representations (e.g., posterior parietal areas, the cerebellum), in the current study, we introduced the parameter “percentages of correct responses in both anticipation conditions” (*M* = 67.39%, *SD* = 6.17) as a parameter to the contrast *Tennis and Volleyball Anticipation* > *Tennis and Volleyball Observation* for all 31 participants. The ROI analysis revealed that in all participants irrespective of the expertise sport a better anticipation performance in both anticipation conditions was correlated with stronger activation of the SPL (5 Ci, 7 P) and Lobule VIIIa and Crus I of the cerebellum (cf. Figure 4A, for a summary of the results, see Table 2).

**Table 2:** **Brain areas showing stronger activation as a function of the number of correct responses in tennis and volleyball serve anticipation conditions when contrasting the anticipation of serves in both sports with the ball bouncing conditions in both sports in all 31 participants**.

	**L/R**	***X***	***Y***	***Z***	***t*-value**	**SUIT**
**TENNIS AND VOLLEYBALL ANTICIPATION > TENNIS AND VOLLEYBALL OBSERVATION**						
SPL (5 Ci)	L	−15	−34	44	2.87	
SPL (7 P)	R	27	−46	50	2.84	
Cerebellum, Lobule VIIIa	R	32	−54	−49	3.28	✓
Cerebellum, Crus I	L	−4	−78	−27	2.08	✓

*MNI coordinates, p < 0.05, FWE-corrected, ROI analysis, ROI masks thresholded at 50%, for all ROI masks used for this analysis see Section Data Analysis at page 4*.

To identify brain activation correlated with the anticipation performance in the expertise sport of the participants, we introduced the percentages of correct responses in the expert anticipation condition (*M* = 70.06%, *SD* = 9.94) as a parameter to the contrast *Expertise Anticipation* > *Expertise Observation* for all 31 participants. A ROI analysis of the influence of the parameter on this contrast resulted in a performance-related increase of activation in the SPL (5 Ci) (cf. Figure 4B, for a summary of the results, see Table 3).

**Table 3:** **Brain areas showing stronger activation as a function of the number of correct responses in serve anticipation in the expertise sport when contrasting the anticipation of serves in the respective expertise sport with the ball bouncing condition in the corresponding expertise sport in all 31 participants**.

	**L/R**	***X***	***Y***	***Z***	***t*-value**	**SUIT**
**EXPERTISE ANTICIPATION > EXPERTISE OBSERVATION**						
SPL (5 Ci)	L	−15	−34	44	2.27	

*MNI coordinates, p < 0.05, FWE-corrected, ROI analysis, ROI masks thresholded at 50%, for all ROI masks used for this analysis see Section Data Analysis at page 4*.

## Discussion

We hypothesized that the anticipation of action effects in sport experts is associated with an increased activation in areas of the AON and in the cerebellum as these areas are discussed to play a crucial role in action observation, anticipation and in motor control (Gazzola and Keysers, 2009; Wright et al., 2010, 2011; Zentgraf et al., 2011; Abreu et al., 2012; Stadler et al., 2012; Avenanti et al., 2013; Bishop et al., 2013; Diersch et al., 2013; Balser et al., 2014). Furthermore, we expected a linear performance-dependent and expertise-related activation increase in AON areas which are primarily suggested to contain perceptual-motor representations during the anticipation task. On the behavioral level, the present findings replicated previous research that both expert groups outperformed the respective novice groups with respect to the number of correct responses concerning the early anticipation of an opponent's action effects. Thus, our results are in line with numerous published reports that demonstrated an expertise effect for the anticipation performance on a behavioral level (see, for a review, Williams et al., 2011). Additionally, the analysis of the response times in both expertise groups revealed a faster response of the experts in their respective expertise sport. Such a result has already been shown by Williams et al. (2002) in a study with tennis experts and novices. Regarding to the authors, the faster anticipation of the experts in the expertise sport is a further indication for superior anticipatory abilities. We are therefore confident that we can interpret the current fMRI results as a result of specific expertise differences.

Regarding the neural level, three main findings of the present study provide support for our hypotheses. First, we show that experts across two different expertise groups in volleyball and tennis revealed an increased activation within broad areas of the AON, more precisely within the preSMA, the SPL, as well as within broad sections of the cerebellum during anticipation of action effects of an opponent in the sport in which they had expertise. Second, we show that irrespective of expertise the percentage of correct responses in the anticipation conditions is associated with stronger activation in the SPL (Areas 5 Ci, 7 P) as well as in the Lobule VIIIa and Crus I of the cerebellum. Third and most important, particularly in motor experts, increasing activation of the superior parietal cortex (5 Ci) co-varies systematically with the anticipation performance during the task.

The present results underpin the notion that the AON, especially posterior parietal sites and the cerebellum are mandatory for the anticipation of action effects and were influenced by the acquired motor skills of the observer (Wright et al., 2010, 2011; Bishop et al., 2013; Balser et al., 2014). The new striking contributions to the literature are that neural activation within the cerebellum and the SPL is linearly correlated with an expert's anticipation performance and that these effects also occur when using a very conservative experimental condition as both expertise groups saw the same stimuli. Customarily, in the field of action anticipation, expertise studies compare the performance of experts in a specific domain with novices who do not exhibit any specific anticipation expertise. The present study differs markedly from prior studies. Here we compared two expert groups who both were defined by extraordinary anticipation skills in their specific domain of expertise but who were at the same time novices for the other sports. This comparison allows us to study very specific effects concerning the individual motor experience in a within-subject design. Therefore, these data conclusively support the notion that the AON as well as cerebellar areas responded to the stimuli in a way that depends on the observer's domain-specific motor expertise what suggests that anticipation of action effects recruits areas of this network depending on the very specific representations of the observed movement in the subject's personal motor repertoire. The following sections will discuss these findings and their implications in more detail.

### Perceptual and motor excellence is linked to activation within the AON during effect anticipation

The process of an appropriate reaction to an opponent's action outcome comprises several computations in the motor system. First, one is requested to accurately predict the consequence of the observed motor action. Second, one has to combine these predictions with the own body state. Third one has then to plan a reaction to the opponent's behavior. Especially the function of an accurate prediction corresponds well to activation within regions of the AON (Wright and Jackson, 2007; Gazzola and Keysers, 2009; Urgesi et al., 2010; Wright et al., 2010, 2011; Zentgraf et al., 2011; Abreu et al., 2012; Stadler et al., 2012; Avenanti et al., 2013; Bishop et al., 2013; Diersch et al., 2013; Balser et al., 2014). The present data revealed that both expert groups outperformed the respective novice groups with respect to the number of correct responses. These effects are accompanied on the neural level with an increased activation within the SMA, the SPL, as well as within sections of the cerebellum what is in line with broad body of literature (Stadler et al., 2011; Wright et al., 2011; Abreu et al., 2012; Bishop et al., 2013; Balser et al., 2014). For example, a recent study by Balser et al. (2014) demonstrated that tennis experts performed better than novices on different tennis anticipation tasks, with the experts showing stronger neural activation in areas of the AON, namely, the superior parietal lobe, the intraparietal sulcus, the inferior frontal gyrus, and the cerebellum. Similarly, Bishop et al. (2013) showed an expertise effect by demonstrating increased cerebellar, cingular and basal ganglia activation for experts during the prediction of the opponent's actions. The findings of Bishop and colleagues and the present results show that the perceptual, motor and cognitive superiority of an expert is clearly linked to increased activation within areas involved in action perception and motor control. On this background, a parametric analysis of the present data revealed that the activation within the Area 5 Ci of the superior parietal activation site and Lobule VIIIa and Crus I of the cerebellum are linearly associated with the anticipation performance irrespective of motor expertise. When comparing effect anticipation in the expertise sport with the observation condition, the parametric relationship between the performance and neural activation still holds for the superior parietal site (Area 5 Ci). This differential involvement of the SPL reflects the performance of motor experts in the expertise-related anticipation task: a better anticipation performance in the expertise sport is related to an increased activation within this region.

### Parietal contributions to the anticipation of action effects

Regarding the posterior parietal cortex, researchers have revealed over the last decade that this area is not only related to higher-order sensory analysis but also plays an important role in motor control (Fogassi and Luppino, 2005; Vesia et al., 2006). For example, it is crucial for visually guided actions. The activation of the SPL is related to the on-line control for reaching, grasping or pointing movements (Grafton et al., 1992, 1996; Culham and Valyear, 2006). In this regard, it was demonstrated that with the growing accuracy demands of an executed aiming task, neural activity within this area increases in line with increased visuomotor processing demands (Winstein et al., 1997; Fiehler et al., 2008), which suggests that the increased activation of posterior parietal sites like the SPL reflects the importance of the target representation when the planned movement comprises a target region. A further functional issue of the SPL is the storage of internal models and action representations which are mandatory for action prediction (Winstein et al., 1997; Wolpert et al., 1998a; Miall, 2003; Rizzolatti and Matelli, 2003).

In the present study, we found a performance-related activation increase in the medial section of the SPL irrespective of motor expertise as well as when comparing the anticipation of serves in the respective expertise sport with the ball bouncing condition in the corresponding expertise sport. Thus, the SPL activation is strongly related to anticipation performance in each participant and depends on the observer's domain-specific motor repertoire. It is likely that the activation within this area, which is functionally associated with visuomotor representations and motor prediction, accompanies the higher-order perceptual and anticipation skills seen in elite athletes, particularly in fast ball sports like tennis and volleyball where a precise coding of spatial information with respect to a target is required. It can be argued that in the present anticipation task, motor expertise seems to enhance the use of these specific internal perceptual-motor representations which are built up through years of training in a certain field of sports.

Another line of research demonstrated activation in the SPL when participants had to initiate movements based on prior expectations (Imamizu and Kawato, 2008). More precisely, it was concluded that the SPL associates contextual information with an appropriate internal model processed in the cerebellum to predict the consequences of an action. It can be argued that experts build up a very specific representation of the contextual framework, such as the opponent's position and its surrounding, which is strongly depending on the type of sports. Within this framework, several researchers have shown that experts improve their anticipation performance when they are provided with contextual, game-related information (Crognier and Féry, 2005; McPherson and MacMahon, 2008; McRobert et al., 2011). Thus, an alternative explanation for the SPL activation pattern within the present study could be that experts use such specific contextual information during the anticipation of their opponent's behavior what is particularly reflected by the expertise- and performance-related increase of the SPL activation.

### Cerebellar contributions to the anticipation of action effects

As for posterior parietal areas, neurophysiological and computational studies have demonstrated the cerebellum as a principal brain structure for the storage of internal forward models that predict action outcomes and therefore support predictive motor control (Wolpert et al., 1998b; Imamizu et al., 2000; Bastian, 2006; Miall and King, 2008; Synofzik et al., 2008). We found that besides the neural activation within the SPL the activation within the cerebellum co-varies systematically with the anticipation performance irrespective of the specific motor expertise. These results are nicely in line with our previous data which reported that activation of parts of the cerebellum co-varies with the anticipation performance irrespective of the motor expertise (Balser et al., 2014). However, the present study expands this finding as this relationship also holds for a within-subject design with two expert groups who both were defined by extraordinary anticipation skills in their specific domain of expertise but who were at the same time novices for the other sports.

It is argued that the cerebellum might house the so-called forward models (Wolpert et al., 1998b; Imamizu et al., 2000; Bastian, 2006; Miall and King, 2008; Synofzik et al., 2008) that are predictive on their part and, therefore, estimate the anticipated sensory outcome of an action (Miall and Wolpert, 1996; Wolpert and Flanagan, 2001). A recent study in cats, for example, showed that neuronal discharge in the lateral cerebellum predicts the motion of a moving external target (Cerminara et al., 2009). These data suggest a connection between a forward model, which predicts the sensory consequences of one's own actions, and a model that could predict the actions of others which has its neural substrate in the cerebellum. The authors reasoned that the measured neural discharge might be used in a predictive capacity for target interception. Extrapolating these data to the present results, it can be suggested that in both, volleyball and tennis, participants are required to predict the effect of an opponent's motion on ball trajectory (Yarrow et al., 2009) by using forward models that allow a rapid processing of incoming sensory stimuli. This offers the acting individual a clear advantage in producing a quick motor response which is mandatory in both sports.

### Differential involvement of cerebellum and SPL during action anticipation

The present results demonstrate a differential involvement of cerebellar and superior parietal areas. Whereas the cerebellum shows a performance dependent activation increase irrespective from expertise, the superior parietal cortex shows a performance and expertise related activation increase. Thus, it seems reasonable to conceive a differential involvement of both structures in action anticipation. Imamizu and Kawato (2008) argued that the SPL associates contextual information with an appropriate internal model located in the cerebellum to predict the consequences of an action. Furthermore, it has been suggested that internal models are acquired in the cerebellum and top–down context information from the SPL to the cerebellum contributes to predictive switching between internal models (Imamizu and Kawato, 2008). We suggest that the expertise and performance dependent activation within the SPL reflects the processing of domain-specific contextual information (e.g., using a racket or not to hit the ball) and leads specifically to increased resonance in the expert's SPL. The activation of the cerebellum, however, reflects the usage of a predictive internal model to solve the anticipation task which is required for both anticipation tasks in the present setting.

### Potential limitations

In the present study, we examined scenes from fast ball games that require quick responses under time pressure. The anticipation of respective action effects in tennis and volleyball include short time windows that are typical for fast ball sports but different to everyday anticipation problems. In our case, participants had to predict distal action effects of an opponent that were at the same time relevant for a selection of own motor responses. The present data, therefore, might not hold for all possible types of anticipation, like the anticipation of in-animated events (Schubotz, 2007) or the anticipation during serial prediction tasks and arbitrary stimulus-response mappings (Wolfensteller et al., 2004).

One possible flaw in the interpretation of the present data is related to the performance-related activation increase in the SPL we found when comparing the anticipation of serves with the ball bouncing condition within the respective expertise sport as well as when comparing both conditions irrespective of motor expertise.

To control for effects due to visual stimulation and the observation of biological movements, we contrasted the anticipation conditions with observation only conditions without an explicit instruction for anticipation. Although the anticipation and the observation only conditions were comparable concerning the depicted models, the sports hall background, the perspective of the camera and the fact that all conditions involved the observation of biological movements that included a ball, both conditions possibly resulted in differential attentional demands. Therefore, we cannot preclude that the posterior parietal activation is also associated with attention-related processes, as the posterior parietal areas has been shown to be involved in directing spatial attention and in disengaging and maintaining attention to visual and tactile stimuli (Posner et al., 1984; Pardo et al., 1991; Corbetta et al., 1993; Halligan et al., 2003; for a review, see Rushworth et al., 2003). However, the comparison of high expertise effect anticipation with low expertise effect anticipation [(*Expertise Anticipation* > *Expertise Observation*) > (*Novice Anticipation* > *Novice Observation*)] revealed activation in the SPL as well. In this contrast, before both anticipation conditions were compared, they were contrasted with the respective observation only condition in a first step. As prospective attention-related differences between the anticipation and the observation only conditions were supposed to be comparable in high and low expertise sport, the influence of the observation only condition concerning attention-related phenomena was minimized. Thus, activation differences in the SPL cannot be assigned to differences in the attention demand between the anticipation and the observation only conditions but to anticipation processes that are modulated by expertise. Furthermore, it has also been argued that the SPL is not the key structure in disengaging attention and further attention-related processes (Corbetta et al., 1995; Rizzolatti et al., 1997; Friedrich et al., 1998). In fact, Rizzolatti et al. (1997) state that the SPL plays a decisive role in the processing of sensory and motor signals in the context of somatosensory integration. Additionally, we examined the activation in the FEF for the comparison the expertise and the novice anticipation condition ((*Expertise Anticipation* > *Expertise Observation*) > (*Novice Anticipation* > *Novice Observation*)) in a *post-hoc* analysis. The FEF has been shown to be involved in attention-related eye movements (Bosch et al., 2013; Squire et al., 2013) and in the allocation of attention in a visual scene (Corbetta and Shulman, 2002; Heinen et al., 2013; Ronconi et al., 2014). The fact that we found no differences in the activation of the FEF between both anticipation conditions indicates that the stronger activation in the expertise sport is not due to differences in attention-related processes.

## Conclusion

We conclude that neural activation within several sections of the AON, especially within the superior parietal as well as within the cerebellar cortex, is associated with action anticipation performance in sport experts. The present data suggest that the AON, including cerebellar areas, responded to the stimuli in a way that depends on the domain-specific representation of the observed action in the subject's personal motor repertoire as well as on the achievement in this task. The present results extend the literature and findings from our previous work by using a very conservative design to show that especially neural activation within the SPL and the cerebellum is linearly associated with the task achievement, irrespective of the specific expertise. For the SPL, this relationship holds when an expert performs a domain-specific anticipation task. We consider that this activation pattern reflects that posterior parietal as well as cerebellar areas are the predominant brain sites that supposed to be involved in fast motor prediction. We suggest that the SPL reflects the processing of domain-specific contextual information (e.g., using a racket or not to hit the ball) and the activation of the cerebellum reflects the usage of a predictive internal model to solve the anticipation task.

### Conflict of interest statement

The authors declare that the research was conducted in the absence of any commercial or financial relationships that could be construed as a potential conflict of interest.

## References

[B1] Abernethy B. Gill D. P. Parks S. L. Packer S. T. (2001). Expertise and the perception of kinematic and situational probability information. Perception 30, 233–252 10.1068/p2872 11296504

[B2] Abernethy B. Russell D. G. (1987). The relationship between expertise and visual search strategy in a racquet sport. Hum. Mov. Sci. 6, 283–319

[B3] Abreu A. M. Macaluso E. Azevedo R. T. Cesari P. Urgesi C. Aglioti S. M. (2012). Action anticipation beyond the action observation network: a functional magnetic resonance imaging study in expert basketball players. Eur. J. Neurosci. 35, 1646–1654 10.1111/j.1460-9568.2012.08104.x 22541026

[B4] Aglioti S. M. Cesari P. Romani M. Urgesi C. (2008). Action anticipation and motor resonance in elite basketball players. Nat. Neurosci. 11, 1109–1116 10.1038/nn.2182 19160510

[B5] Avenanti A. Annella L. Candidi M. Urgesi C. Aglioti S. M. (2013). Compensatory plasticity in the action observation network: virtual lesions of STS enhance anticipatory simulation of seen actions. Cereb. Cortex 23, 570–580 10.1093/cercor/bhs040 22426335

[B6] Balser N. Lorey B. Pilgramm S. Stark R. Bischoff M. Zentgraf K. (2014). Prediction of human actions: expertise and task-related effects on neural activation of the action observation network. Hum. Brain Mapp. 35, 4016–4034 10.1002/hbm.22455 24453190 PMC6869237

[B7] Bastian A. J. (2006). Learning to predict the future: the cerebellum adapts feedforward movement control. Curr. Opin. Neurobiol. 16, 645–649 10.1016/j.conb.2006.08.016 17071073

[B8] Bishop D. T. Wright M. J. Jackson R. C. Abernethy B. (2013). Neural bases for anticipation skill in soccer: an FMRI study. J. Sport Exerc. Psychol. 35, 98–109 23404883 10.1123/jsep.35.1.98

[B9] Bosch S. E. Neggers S. F. W. van der Stigchel S. (2013). The role of the frontal eye fields in oculomotor competition: image-guided TMS enhances contralateral target selection. Cereb. Cortex 23, 824–832 10.1093/cercor/bhs075 22455840

[B10] Buccino G. Vogt S. Ritzl A. Fink G. R. Zilles K. Freund H.-J. (2004). Neural circuits underlying imitation learning of hand actions: an event-related fMRI study. Neuron 42, 323–334 10.1016/S0896-6273(04)00181-3 15091346

[B11] Calvo-Merino B. Grèzes J. Glaser D. E. Passingham R. E. Haggard P. Gre J. (2006). Seeing or doing? Influence of visual and motor familiarity in action observation. Curr. Biol. 16, 1905–1910 10.1016/j.cub.2006.07.065 17027486

[B12] Cañal-Bruland R. van Ginneken W. F. van der Meer B. R. Williams A. M. (2011). The effect of local kinematic changes on anticipation judgments. Hum. Mov. Sci. 30, 495–503 10.1016/j.humov.2010.10.001 21239078

[B13] Caspers S. Zilles K. Laird A. R. Eickhoff S. B. (2010). ALE meta-analysis of action observation and imitation in the human brain. Neuroimage 50, 1148–1167 10.1016/j.neuroimage.2009.12.112 20056149 PMC4981639

[B14] Cerminara N. L. Apps R. Marple-Horvat D. E. (2009). An internal model of a moving visual target in the lateral cerebellum. J. Physiol. 587, 429–442 10.1113/jphysiol.2008.163337 19047203 PMC2670054

[B15] Corbetta M. Miezin F. M. Shulman G. L. Petersen S. E. (1993). A PET study of visuospatial attention. J. Neurosci. 13, 1202–1226 8441008 10.1523/JNEUROSCI.13-03-01202.1993 PMC6576604

[B16] Corbetta M. Shulman G. L. (2002). Control of goal-directed and stimulus-driven attention in the brain. Nat. Rev. Neurosci. 3, 201–215 10.1038/nrn755 11994752

[B17] Corbetta M. Shulman G. L. Miezin F. M. Petersen S. E. (1995). Superior parietal cortex activation during spatial attention shifts and visual feature conjunction. Science 270, 802–805 7481770 10.1126/science.270.5237.802

[B18] Crognier L. Féry Y.-A. (2005). Effect of tactical initiative on predicting passing shots in tennis. Appl. Cogn. Psychol. 19, 637–649 10.1002/acp.1100

[B19] Cross E. S. Hamilton A. F. C. de Kraemer D. J. M. Kelley W. M. Grafton S. T. (2009). Sensitivity of the action observation network to physical and observational learning. Cereb. Cortex 19, 315–326 10.1093/cercor/bhn083 18515297 PMC2638791

[B20] Culham J. C. Valyear K. F. (2006). Human parietal cortex in action. Curr. Opin. Neurobiol. 16, 205–212 10.1016/j.conb.2006.03.005 16563735

[B21] Diedrichsen J. (2006). A spatially unbiased atlas template of the human cerebellum. Neuroimage 33, 127–138 10.1016/j.neuroimage.2006.05.056 16904911

[B22] Diedrichsen J. Balsters J. H. Flavell J. Cussans E. Ramnani N. (2009). A probabilistic MR atlas of the human cerebellum. Neuroimage 46, 39–46 10.1016/j.neuroimage.2009.01.045 19457380

[B23] Diersch N. Mueller K. Cross E. S. Stadler W. Rieger M. Schütz-Bosbach S. (2013). Action prediction in younger versus older adults: Neural correlates of motor familiarity. PLoS ONE 8:e64195 10.1371/journal.pone.0064195 23704980 PMC3660406

[B24] Dimitrova A. de Greiff A. Schoch B. Gerwig M. Frings M. Gizewski E. R. (2006). Activation of cerebellar nuclei comparing finger, foot and tongue movements as revealed by fMRI. Brain Res. Bull. 71, 233–241 10.1016/j.brainresbull.2006.09.015 17113951

[B25] Eickhoff S. B. Stephan K. E. Mohlberg H. Grefkes C. Fink G. R. Amunts K. (2005). A new SPM toolbox for combining probabilistic cytoarchitectonic maps and functional imaging data. Neuroimage 25, 1325–1335 10.1016/j.neuroimage.2004.12.034 15850749

[B26] Fiehler K. Burke M. Engel A. Bien S. Rösler F. (2008). Kinesthetic working memory and action control within the dorsal stream. Cereb. Cortex 18, 243–253 10.1093/cercor/bhm071 17548801

[B27] Fogassi L. Luppino G. (2005). Motor functions of the parietal lobe. Curr. Opin. Neurobiol. 15, 626–631 10.1016/j.conb.2005.10.015 16271458

[B28] Friedrich F. J. Egly R. Rafal R. D. Beck D. (1998). Spatial attention deficits in humans: a comparison of superior parietal and temporal-parietal junction lesions. Neuropsychology 12, 193–207 9556766 10.1037//0894-4105.12.2.193

[B29] Gallagher H. L. Frith C. D. (2004). Dissociable neural pathways for the perception and recognition of expressive and instrumental gestures. Neuropsychologia 42, 1725–1736 10.1016/j.neuropsychologia.2004.05.006 15351623

[B30] Gazzola V. Keysers C. (2009). The observation and execution of actions share motor and somatosensory voxels in all tested subjects: single-subject analyses of unsmoothed fMRI data. Cereb. Cortex 19, 1239–1255 10.1093/cercor/bhn181 19020203 PMC2677653

[B31] Gazzola V. Rizzolatti G. Wicker B. Keysers C. (2007). The anthropomorphic brain: the mirror neuron system responds to human and robotic actions. Neuroimage 35, 1674–1684 10.1016/j.neuroimage.2007.02.003 17395490

[B32] Gläscher J. (2009). Visualization of group inference data in functional neuroimaging. Neuroinformatics 7, 73–82 10.1007/s12021-008-9042-x 19140033

[B33] Grafton S. T. Arbib M. A. Fadiga L. Rizzolatti G. (1996). Localization of grasp representations in humans by positron emission tomography. 2. Observation compared with imagination. Exp. Brain Res. 112, 103–111 8951412 10.1007/BF00227183

[B34] Grafton S. T. Mazziotta J. C. Woods R. P. Phelps M. E. (1992). Human functional anatomy of visually guided finger movements. Brain 115, 565–587 1606482 10.1093/brain/115.2.565

[B35] Halligan P. W. Fink G. R. Marshall J. C. Vallar G. (2003). Spatial cognition: evidence from visual neglect. Trends Cogn. Sci. 7, 125–133 10.1016/S1364-6613(03)00032-9 12639694

[B36] Heinen K. Feredoes E. Weiskopf N. Ruff C. C. Driver J. (2013). Direct evidence for attention-dependent influences of the frontal eye-fields on feature-responsive visual cortex. Cereb. Cortex. [Epub ahead of print]. 10.1093/cercor/bht157 23794715 PMC4193466

[B37] Hubert M. van der Veeken S. (2008). Outlier detection for skewed data. J. Chemometr. 22, 235–246 10.1002/cem.112322920157

[B38] Huys R. Smeeton N. J. Hodges N. J. Beek P. J. Williams A. M. (2008). On the dynamic information underlying visual anticipation skill. Percept. Psychophys. 70, 1217–1234 10.3758/PP.70.7.1217 18927005

[B39] Imamizu H. Kawato M. (2008). Neural correlates of predictive and postdictive switching mechanisms for internal models. J. Neurosci. 28, 10751–10765 10.1523/JNEUROSCI.1106-08.2008 18923050 PMC6671335

[B40] Imamizu H. Miyauchi S. Tamada T. Sasaki Y. Takino R. Pütz B. (2000). Human cerebellar activity reflecting an acquired internal model of a new tool. Nature 403, 192–195 10.1038/35003194 10646603

[B41] McPherson S. L. MacMahon C. (2008). How baseball players prepare to bat: tactical knowledge as a mediator of expert performance in baseball. J. Sport Exerc. Psychol. 30, 755–778 19164840 10.1123/jsep.30.6.755

[B42] McRobert A. P. Ward P. Eccles D. W. Williams A. M. (2011). The effect of manipulating context-specific information on perceptual–cognitive processes during a simulated anticipation task. Brit. J. Psychol. 102, 519–534 10.1111/j.2044-8295.2010.02013.x 21752003

[B43] Miall R. C. (2003). Connecting mirror neurons and forward models. Neuroreport 14, 2135–2137 10.1097/01.wnr.0000098751.87269.77 14625435

[B44] Miall R. C. King D. (2008). State estimation in the cerebellum. Cerebellum 7, 572–576 10.1007/s12311-008-0072-6 18855092 PMC6010151

[B45] Miall R. C. Wolpert D. M. (1996). Forward models for physiological motor control. Neural Networks 9, 1265–1279 12662535 10.1016/s0893-6080(96)00035-4

[B46] Molenberghs P. Cunnington R. Mattingley J. B. (2012). Brain regions with mirror properties: a meta-analysis of 125 human fMRI studies. Neurosci. Biobehav. Rev. 36, 341–349 10.1016/j.neubiorev.2011.07.004 21782846

[B47] Oldfield R. (1971). The assessment and analysis of handedness: the Edinburgh inventory. Neuropsychologia 9, 97–113 5146491 10.1016/0028-3932(71)90067-4

[B48] Pardo J. V. Fox P. T. Raichle M. E. (1991). Localization of a human system for sustained attention by positron emission tomography. Nature 349, 61–64 10.1038/349061a0 1985266

[B49] Pilgramm S. Lorey B. Stark R. Munzert J. Vaitl D. Zentgraf K. (2010). Differential activation of the lateral premotor cortex during action observation. BMC Neurosci. 11:89 10.1186/1471-2202-11-89 20673366 PMC2923156

[B49a] Posner M. I. Walker J. A. Friedrich F. J. Rafal R. D. (1984). Effects of parietal injury on covert orienting of attention. J. Neurosci. 4, 1863–1874 6737043 10.1523/JNEUROSCI.04-07-01863.1984 PMC6564871

[B50] Rizzolatti G. Fogassi L. Gallese V. (1997). Parietal cortex: from sight to action. Curr. Opin. Neurobiol. 7, 562–567 9287198 10.1016/s0959-4388(97)80037-2

[B51] Rizzolatti G. Matelli M. (2003). Two different streams form the dorsal visual system: anatomy and functions. Exp. Brain Res. 153, 146–157 10.1007/s00221-003-1588-0 14610633

[B52] Ronconi L. Basso L. Gori S. Facoetti A. (2014). TMS on right frontal eye fields induces an inflexible focus of attention. Cereb. Cortex 24, 396–402 10.1093/cercor/bhs319 23048022

[B53] Rowe R. M. McKenna F. P. (2001). Skilled anticipation in real-world tasks: measurement of attentional demands in the domain of tennis. J. Exp. Psychol. Appl. 7, 60–67 10.1037/1076-898X.7.1.60 11577620

[B54] Rushworth M. F. S. Johansen-Berg H. Göbel S. M. Devlin J. T. (2003). The left parietal and premotor cortices: motor attention and selection. Neuroimage 20, S89–100 10.1016/j.neuroimage.2003.09.011 14597301

[B55] Savelsbergh G. J. P. Williams A. M. van der Kamp J. Ward P. (2002). Visual search, anticipation and expertise in soccer goalkeepers. J. Sports Sci. 20, 279–287 10.1080/026404102317284826 11999482

[B56] Schmahmann J. D. Macmore J. Vangel M. (2009). Cerebellar stroke without motor deficit: clinical evidence for motor and non-motor domains within the human cerebellum. Neuroscience 162, 852–861 10.1016/j.neuroscience.2009.06.023 19531371 PMC2763197

[B57] Schubotz R. (2007). Prediction of external events with our motor system: towards a new framework. Trends Cogn. Sci. 11, 211–218 10.1016/j.tics.2007.02.006 17383218

[B58] Singer R. N. Cauraugh J. H. Chen D. Steinberg G. M. (1996). Visual search, anticipation, and reactive comparisons between highly-skilled and beginning tennis players. J. Appl. Sport Psychol. 8, 9–26

[B59] Smith S. M. Jenkinson M. Woolrich M. W. Beckmann C. F. Behrens T. E. J. Johansen-Berg H. (2004). Advances in functional and structural MR image analysis and implementation as FSL. Neuroimage 23, 208–219 10.1016/j.neuroimage.2004.07.051 15501092

[B60] Sokolov A. A. Gharabaghi A. Tatagiba M. S. Pavlova M. (2010). Cerebellar engagement in an action observation network. Cereb. Cortex 20, 486–491 10.1093/cercor/bhp117 19546157

[B61] Squire R. F. Noudoost B. Schafer R. J. Moore T. (2013). Prefrontal contributions to visual selective attention. Annu. Rev. Neurosci. 36, 451–466 10.1146/annurev-neuro-062111-150439 23841841

[B62] Stadler W. Ott D. V. M. Springer A. Schubotz R. I. Schütz-Bosbach S. Prinz W. (2012). Repetitive TMS suggests a role of the human dorsal premotor cortex in action prediction. Front. Hum. Neurosci. 6:20 10.3389/fnhum.2012.00020 22363279 PMC3282473

[B63] Stadler W. Schubotz R. I. von Cramon D. Y. von Springer A. Graf M. Prinz W. (2011). Predicting and memorizing observed action: differential premotor cortex involvement. Hum. Brain Mapp. 32, 677–687 10.1002/hbm.20949 20225220 PMC6870275

[B64] Synofzik M. Lindner A. Thier P. (2008). The cerebellum updates predictions about the visual consequences of one's behavior. Curr. Biol. 18, 814–818 10.1016/j.cub.2008.04.071 18514520

[B65] Urgesi C. Maieron M. Avenanti A. Tidoni E. Fabbro F. Aglioti S. M. (2010). Simulating the future of actions in the human corticospinal system. Cereb. Cortex 20, 2511–2521 10.1093/cercor/bhp292 20051359

[B66] Urgesi C. Savonitto M. M. Fabbro F. Aglioti S. M. (2011). Long- and short-term plastic modeling of action prediction abilities in volleyball. Psychol. Res. 76, 542–560 10.1007/s00426-011-0383-y 22045443

[B67] Vesia M. Monteon J. A. Sergio L. E. Crawford J. D. (2006). Hemispheric asymmetry in memory-guided pointing during single-pulse transcranial magnetic stimulation of human parietal cortex. J. Neurophysiol. 96, 3016–3027 10.1152/jn.00411.2006 17005619

[B68] Williams A. M. Ford P. R. Eccles D. W. Ward P. (2011). Perceptual-cognitive expertise in sport and its acquisition: implications for applied cognitive psychology. Appl. Cogn. Psychol. 25, 432–442 10.1002/acp.1710

[B69] Williams A. M. Huys R. Cañal-Bruland R. Hagemann N. (2009). The dynamical information underpinning anticipation skill. Hum. Mov. Sci. 28, 362–370 10.1016/j.humov.2008.10.006 19081648

[B70] Williams A. M. Ward P. Knowles J. M. Smeeton N. J. (2002). Anticipation skill in a real-world task: measurement, training, and transfer in tennis. J. Exp. Psychol. Appl. 8, 259–270 10.1037/1076-898X.8.4.259 12570100

[B71] Winstein C. J. Grafton S. T. Pohl P. S. Angeles L. (1997). Motor task difficulty and brain activity: investigation of goal-directed reciprocal aiming using positron emission tomography. J. Neurophysiol. 77, 1581–1594 9084621 10.1152/jn.1997.77.3.1581

[B72] Wolfensteller U. Schubotz R. I. von Cramon D. Y. (2004). “What” becoming “where”: functional magnetic resonance imaging evidence for pragmatic relevance driving premotor cortex. J. Neurosci. 17, 10431–10439 10.1523/JNEUROSCI.2641-042004 15548658 PMC6730301

[B73] Wolpert D. M. Flanagan J. R. (2001). Motor prediction. Curr. Biol. 11, 729–732 10.1016/S0960-9822(01)00432-8 11566114

[B74] Wolpert D. M. Goodbody S. J. Husain M. (1998a). Maintaining internal representations: the role of the human superior parietal lobe. Nat. Neurosci. 1, 529–533 10.1038/2245 10196553

[B75] Wolpert D. M. Miall R. C. Kawato M. (1998b). Internal models in the cerebellum. Trends Cogn. Sci. 2, 338–347 21227230 10.1016/s1364-6613(98)01221-2

[B76] Worsley K. (2007). Random field theory, Chapter 18, in Statistical Parametric Mapping: The Analysis of Functional Brain Images, eds Friston K. Ashburner J. Kiebel S. Nichols T. Penny W. (Amsterdam: Academic Press), 232–236

[B77] Wright M. Bishop D. Jackson R. Abernethy B. (2011). Cortical fMRI activation to opponents' body kinematics in sport-related anticipation: expert-novice differences with normal and point-light video. Neurosci. Letters 500, 216–221 10.1016/j.neulet.2011.06.045 21741450

[B78] Wright M. J. Bishop D. T. Jackson R. C. Abernethy B. (2010). Functional MRI reveals expert-novice differences during sport-related anticipation. Neuroreport 21, 94–98 10.1097/WNR.0b013e328333dff2 20051784

[B79] Wright M. J. Jackson R. C. (2007). Brain regions concerned with perceptual skills in tennis: an fMRI study. Int. J. Psychophysiol. 63, 214–220 10.1016/j.ijpsycho.2006.03.018 16797757

[B80] Yarrow K. Brown P. Krakauer J. W. (2009). Inside the brain of an elite athlete: the neural processes that support high achievement in sports. Nat. Rev. Neurosci. 10, 585–596 10.1038/nrn2672 19571792

[B81] Zentgraf K. Munzert J. Bischoff M. Newman-Norlund R. D. (2011). Simulation during observation of human actions-theories, empirical studies, applications. Vision Res. 51, 827–835 10.1016/j.visres.2011.01.007 21277318

