# Supplementary material for: The influence of expertise on brain activation of the action observation network during anticipation of tennis and volleyball serves
Source: Front Hum Neurosci. 2014 Aug 1;8:568. doi: 10.3389/fnhum.2014.00568 (PMC4117995; doi:10.3389/fnhum.2014.00568)
Supplement: Supplementary file 1 [file DataSheet1.DOCX]

**Table S1:**  Brain areas identified by the comparison of the respective expertise anticipation

condition with the corresponding novice anticipation condition when comparing tennis with

volleyball experts. Each anticipation condition was again contrasted with the ball bouncing

condition of the same sport (*Expertise Anticipation* > *Expertise Observation*) > (*Novice*

*Anticipation* > *Novice Observation*).

|  | L/R | X | Y | Z | *t* value |
| --- | --- | --- | --- | --- | --- |
| S1 | L | -42 | -37 | 47 | 4.32 |
| vPMC | L | -57 | 5 | 29 | 3.15 |
| IPS | L | -36 | -46 | 44 | 3.74 |
| IPS | L | -45 | -43 | 41 | 4.77 |
| Broca Area (BA 44) | L | -51 | 8 | 26 | 4.11 |
| SPL (7P) | L | -36 | -49 | 56 | 3.04 |
| IPL | L | -48 | -46 | 53 | 3.62 |
